# Fly-DPI: database of protein interactomes for *D. melanogaster *in the approach of systems biology

**DOI:** 10.1186/1471-2105-7-S5-S18

**Published:** 2006-12-18

**Authors:** Chung-Yen Lin, Shu-Hwa Chen, Chi-Shiang Cho, Chia-Ling Chen, Fan-Kai Lin, Chieh-Hua Lin, Pao-Yang Chen, Chen-Zen Lo, Chao A Hsiung

**Affiliations:** 1 Division of Biostatistics and Bioinformatics, National Health Research Institutes. No. 35 Keyan Rd. Zhunan, Miaoli County 350, Taiwan; 2 Institute of Information Science, Academia Sinica, No. 128 Yan-Chiu-Yuan Rd., Sec. 2, Taipei 115, Taiwan; 3 Institute of Fishery Science, National Taiwan University, No. 1, Sec 4, Roosevelt Road, Taipei, 10617, Taiwan; 4 Stem Cell/Regenerative Medicine Program, Genomics Research Center, Academia Sinica., No. 128 Yan-Chiu-Yuan Rd., Sec. 2, Taipei 115, Taiwan

## Abstract

**Background:**

Proteins control and mediate many biological activities of cells by interacting with other protein partners. This work presents a statistical model to predict protein interaction networks of *Drosophila melanogaster *based on insight into domain interactions.

**Results:**

Three high-throughput yeast two-hybrid experiments and the collection in FlyBase were used as our starting datasets. The co-occurrences of domains in these interactive events are converted into a probability score of domain-domain interaction. These scores are used to infer putative interaction among all available open reading frames (ORFs) of fruit fly. Additionally, the likelihood function is used to estimate all potential protein-protein interactions.

All parameters are successfully iterated and MLE is obtained for each pair of domains. Additionally, the maximized likelihood reaches its converged criteria and maintains the probability stable. The hybrid model achieves a high specificity with a loss of sensitivity, suggesting that the model may possess major features of protein-protein interactions. Several putative interactions predicted by the proposed hybrid model are supported by literatures, while experimental data with a low probability score indicate an uncertain reliability and require further proof of interaction.

Fly-DPI is the online database used to present this work. It is an integrated proteomics tool with comprehensive protein annotation information from major databases as well as an effective means of predicting protein-protein interactions. As a novel search strategy, the ping-pong search is a naïve path map between two chosen proteins based on pre-computed shortest paths. Adopting effective filtering strategies will facilitate researchers in depicting the bird's eye view of the network of interest. Fly-DPI can be accessed at .

**Conclusion:**

This work provides two reference systems, statistical and biological, to evaluate the reliability of protein interaction. First, the hybrid model statistically estimates both experimental and predicted protein interaction relationships. Second, the biological information for filtering and annotation itself is a strong indicator for the reliability of protein-protein interaction. The space-temporal or stage-specific expression patterns of genes are also critical for identifying proteins involved in a particular situation.

## Background

In most cases, proteins are the way that genes exert their function. These macromolecules mediate their functions by forming complicated and interconnected networks that are flexible and dynamic. For instance, more than 200 cell types are identified in the human body. These cells use the same genome content, but different scenarios for their performance. In another case, living organisms have developed various survival tactics protein interactions against nearly all kinds of stresses to persist and to flourish in a changing world. Clarifying the protein-protein interaction network is essential to understanding cellular processes, explaining its prominence as a major field in the post-genomic era. Elucidating protein interacting partnerships may help annotate unknown proteins and provide further insight into biological networks.

Various experimental strategies are available for identifying protein interactions [1]. Among which, expressing open-reading frame sequences as recombinant fusion proteins and studying their pair-wise interactions is an effective strategy. Yeast two-hybrid (Y2H) is the representative means of doing so. Another experimental strategy purifies and analyzes the protein complex using proteomic technology. These strategies can complement each other. While conducive for high-throughput technology, the yeast two-hybrid system has been used in bacteria, yeast, worms, flies and more recently, in mice and humans [2-10]. These works enable us to systematically characterize physical protein-protein interactions. Although the efficiency of the yeast two-hybrid system is attractive to biologists, the high false positive rate of the assay is a serious limitation, thus requiring other validating approaches before using these data. Therefore, statistical models are introduced to systematically eliminate unsatisfactory results [11,12]. Wojcik *et al. *[13] predict protein interactions based on a large scale "reference" interaction map that includes interaction domain information. The use of domain information improves the performance from using sequences solely, that suggests the domain-based approach. Nevertheless, statistical models alone might not persuade biologists. Biological filters, e.g., spatial and temporal information, may provide a rationale for each interaction to more thoroughly understand the dynamic cellular environment.

The protein-protein interaction network is naturally complex. Visualization tools are the most effective means of obtaining a global view of a protein network. Several analytical approaches and visualization systems can depict the interaction map. BIND [14] incorporates a map viewer called SPREY, which solely generates maps by single IDs, in which neither aliases are allowed nor gene annotation attached. JDIP [15], a stand-alone Java application for DIP, functions similarly. Other network viewing systems, such as VisAnt [16], Osprey [17] InterViewer3[18], Pajek [19], or Tulip [20], lack detailed annotation. Constructing a protein network map often becomes chaotic in that numerous nodes and edges are crowded within a limited window screen. Optimizing a clear network display and maintaining useful information would allow researchers to identify their target of interest.

*Drosophila melanogaster *(the fruit fly) has long been a highly useful model organism. By taking advantage of the large body of publicly available biological information, this study constructed an on-line database, Fly-DPI, to present the protein-protein interaction network of flies. Like previously published computational works on protein-protein interaction predictions [11,21-26], our prediction system was based on the concept of domain-based predictions. Information from GO [27], KEGG [28], GenBank, FlyBase [29], and Unigene [30] provide the biological filters and annotations. The model proposed in this study is a hybrid of the association method and maximum likelihood estimation (MLE) method and was applied to estimate the reliability of each experimental protein interaction and also to predict novel protein interactions.

Fly-DPI serves as a user-friendly interface integrated with graphical networking maps based on our previous work [31]. With its abundant biological information as searching filters, annotation tools and statistical estimates to access the reliability of each interaction, the Fly-DPI can provide protein network maps of *D. melanogaster *at specific spatiotemporal stages, composed of experimental and inferred interaction data. The arrangement of nodes and edges in an interacting network is optimized to reduce overlaps and crossover. Patterns and colors of connecting edges are used to indicate various strengths of interactions based on the association measures and different centering node they originate from. Starting from a query result table, proteins of interest can be explored through its interacting partners in the map, and the interaction neighborhood can be extended up to three levels. A new approach, ping-pong search, opens up new avenues for easily acquiring additional putative path maps as segments of a biological pathway. In summary, the Fly-DPI is a system for predicting protein-protein interactions based on known protein-protein interaction data, integrated with complete and useful protein annotation information from major databases. Fly-DPI is designed to explore in-depth potential protein interaction relationships, and provides reliability estimates for protein-protein interactions for both experimental and inferred interaction, which can be used to build a protein interaction network and predict novel interactions. This system offers an important method of studying fly and other species to expand our knowledge in biological and medical studies.

## Graphical view of the fly-DPI

### System implementation

To introduce a user-friendly graphical interface, a database of protein interactomes for *D. melanogaster *(fly-DPI) was constructed with the LAMP system (Linux Mandrake 10, Apache 2.0, MySQL 4.0, and PHP 4.0) and the GD library was used for dynamic image creation.

### Searching protein networks of interest

The general search interface of Fly-DPI allows for a search by ORF name, gene loci, RNA transcripts, FlyBase gene index, GO IDs, KEGG metabolism, EC number, or full-text searches with keywords. Two sets of data pools can be searched. The first is the high-confidence network based on the interactions defined by Giot *et al*. [4], Stanyon *et al*. [33], and Formstecher *et al*. [32]; putative interactions inferred from the high-confidence datasets are also included. The second is all the interactions collected in the Fly-DPI, and comprises the total interaction datasets from previously described experiments, and the interactions inferred from the total datasets. The main GO categories, including biological processes, cellular components and molecular functions, a combination of all of the above are used for query filtering. Users can also confine search queries to proteins located in a specific chromosome. Tissue- or stage-specific expression information of each protein is inferred from the gene expression pattern collected in Unigene and listed in the query options. Currently available options include adult testis, the brain, embryos, head, head-brain and sensory organs, ovaries, salivary glands, testes, the whole body, and the whole embryos. Finally, output format options are available that allow users to retain all or some of the relevant information in the result table. Protein items in the result table are sorted by ORFs, loci, or RNA transcript names.

### Viewing a protein network map

**Figure 1:**
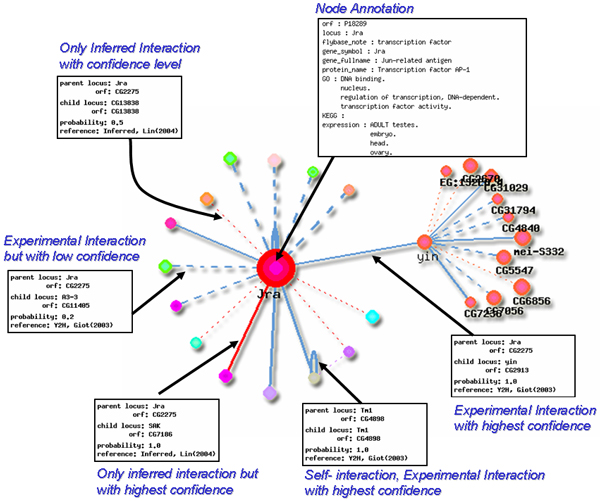
**The map of network**. A map of the experimental and inferred visualized protein interaction networks of *Drosophila melanogaster*. The solid/dotted lines in blue indicate that these interactions exist in experimental results with high/low confidence. The solid and dotted lines in colors (adopted from the color of protein) other than blue respectively depict the putative relations with possibilities equal to one and less than one. Information on edges and nodes is instantly displayed with messaging boxes when the cursor is placed over. The network is expended dynamically with a double-click on the node.

The output table of a query is returned to a new window based on the parameters set in the query form. By selecting an appropriate probability threshold, the interaction map of the selected protein is immediately generated in another new window, or by selecting "exp" to view interactions in the original experimental datasets only. A higher threshold will show those higher confident interactions in the map based on our statistical model. Connecting edge (interaction) color and pattern are used to track back to the starting node (protein) of these interactions when exploring the network and the strength of the interaction using the estimated statistical model. For example, a blue line indicates that the interaction exists in yeast two-hybrid experiments, while other colored lines are based on the node from which these edges have been sent out in previous action. Additionally, a solid line indicates the probability that the interaction approximately equals one, while a dotted line indicates that the interaction has a probability score below 1. In the example case (Jra, P18289 or CG2275, with an interaction probability of 0.4, Fig. 1), the central node is shown as a red node. Moreover, every putative interacting edge of Jra is colored red. Clicking on the node in the current map can extend the interaction network by one interaction level. For example, a user may click on Yin and obtain a new set of Yin-interacting nodes marked in rose.

Information on nodes and edges is displayed in messaging boxes when the cursor is moved over these objects. The "Save" button on the top of the right column permits users to download all the interacting relationships into a standard. csv file which can be opened using Excel or some other spreadsheet program. Meanwhile, image files of the interacting network can be saved in ".bmp" file format. The option of downloading the interaction map in PSI format will soon be provided.

### Ping-Pong -identifying the shortest path between two proteins of interest

**Figure 2:**
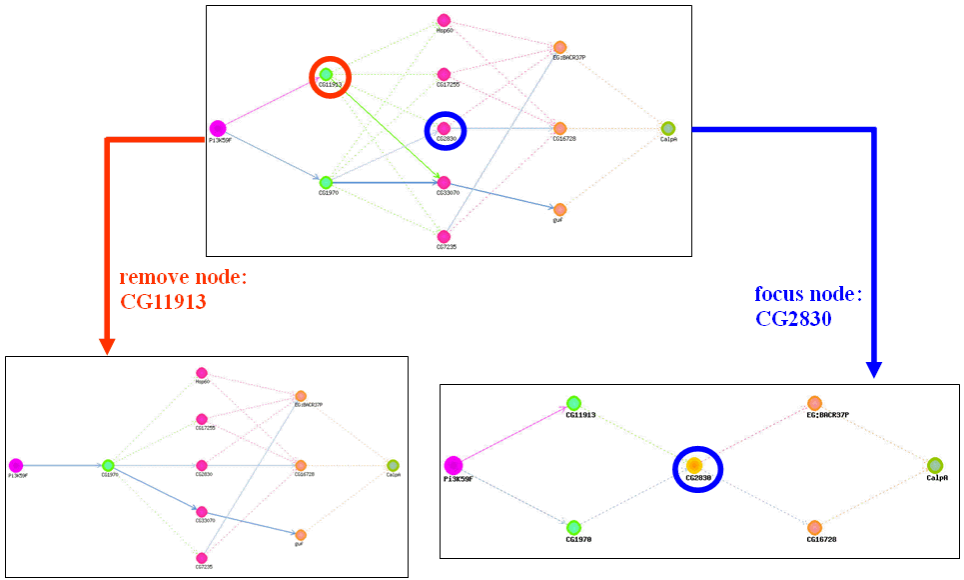
**A snap of Ping-Pong search**. A map of proteins involved in apoptosis generated by the ping-pong search. By clicking on the nodes or lines between two query proteins, the advanced option removed the related paths or confines the paths to the selected nodes or lines.

Fly-DPI provides a innovative search for finding possible connections between two proteins, the **ping-pong search**. After entering ORFs or loci of two target proteins, the interacting networks extend outwards from these two proteins until they reach one another. All possible connections between them in the limiting levels are then shown graphically (Fig. 2). The annotation boxes for edges and nodes work as described previously. Users can download all the interacting relationships in the form of a standard csv file by clicking on "save all path" button. The interconnecting map for the two proteins can then be further refined by clicking on any node, except the two end nodes. The options "focus" and "remove" show up in a new window. The option "focus" picks up a sub-network with paths related to the selected node only, and the option "remove" simply excludes the selected node and the connected paths. If the two proteins are disjointed within the restriction of level setting (up to 5, *viz*, pass through three other nodes from node A to node B), the ping-pong network will recalculate in the background and provides an e-mail notification to users upon their completion. Following the link provided in the email, users can access their query network for one month after the e-mail is sent.

## Result and Discussion

The hybrid model presented here uses legitimate initial association measures to perform heuristic computations for the MLE. A key achievement of the computation with this model is the successful iteration of all parameters (16271 domain interactions derived from total protein interactions, 3,344 domain interactions from high confidence set) and the fact that the obtaining of the MLE for each pair of domains helped the maximized likelihood to reach its converged criteria and maintained the stability of the probability. The proposed method is demonstrated to outperform those methods based on non-informative priors. One model assumption is treating the domain-domain interaction as an independent event. Although most domain-based methods for predicting interaction between proteins also presume that individual domain-domain interactions are independent of the formation of other domain-domain interactions, Han *et al*. [23] proposed a PreSPI system, in which protein-protein interactions are interpreted as the result of interactions of multiple domain pairs or of groups of domains. This study tested both single- and multiple-domain methods using the association and hybrid model. However, no evidence exists supporting the improvement of the multiple-domain method (data not shown).

A hybrid model is applied to the experimental data (23,802 and 2,776 interactions from total and high confidence set, respectively) available from *D. melanogaster *protein interactome. To testify the robustness of the proposed model, the dataset is randomly divided into training and test sets using a ratio of 9:1. To apply Jackknife method to validate the proposed approach, the test set is re-sampled for each iteration of modeling and the average sensitivity and specificity calculated. The probabilities of domain-domain interaction Pr(*D*_*mn *_= 1) are dichotomized according to different thresholds from 0 to 1, as a predictor of protein-protein interactions. The figure showing the supplemental data S1 in additional file 1 illustrates the sensitivity and specificity for each threshold. The Receiver Operating Characteristic (ROC) curve shows that better performance, and greater area below the curve, resulted from using high confidence datasets. The use of high confidence datasets provides more accurate information for estimating the probabilities of domain-domain interactions.

The specificity is higher than for the previous maximum likelihood approach [11,12]. However, arguably the difference in performance among these methods results from the quality and coverage of the datasets used from different organisms. The hybrid model used here achieves high specificity but reduced sensitivity. This suggests that the proposed model may possess some of the key features in parts of protein-protein interactions, though improvements are still necessary, for example using other biological information and more sophisticated statistical methods, and/or increasing coverage of experimental datasets from other sources.

**Figure 3:**
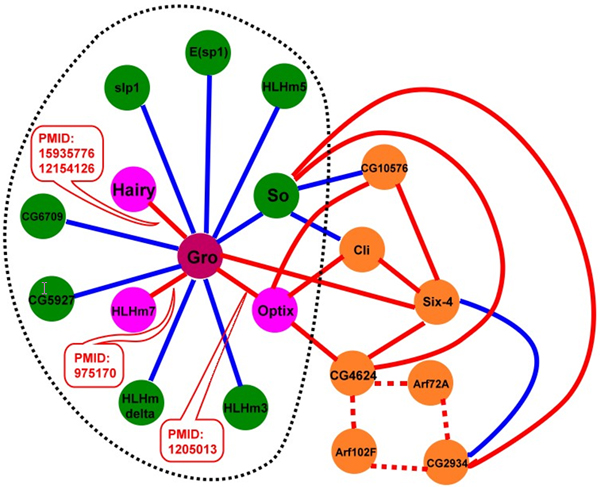
**Interaction network amid Gro and its partners**. Interactions related to development of the Drosophila nervous system. Proof for Gro and HLHm7 [32], Gro and Optix [33], Gro and Six4 [33], and Gro and Hairy [34, 35] was obtained from literatures. The solid and dotted lines in red respectively indicate interactions predicted by our hybrid model with high and low confidence (with a probability of 0.7). The experimental interactions are marked in blue.

Comparing the putative interactions inferred from the high confidence set with those interactions only presented in the literature found that several putative interactions predicted by our hybrid model (Pr = 1) are supported by interaction collected in BIND. The interaction network of Gro protein is used here as an example. As shown in Fig. 3, the solid and dotted lines in red represent the interactions predicted by the hybrid model with both high and low confidence (Pr. = 0.7). Although these interactions do not exist in the original dataset, they were successfully discovered by the proposed prediction model, including the interactions of Gro and HLHm7 [32], Gro and Optix [33], Gro and Six4 [33], Gro and Hairy [34,35]. These proteins are related to the development of peripheral nervous system and fly eye. Since these interactions have been demonstrated by the literatures, it will be interesting to see whether the novel interactions identified in this model with Pr = 1 will be a true interaction or not. The proposed prediction system provides not only putative interacting protein candidates but also reliability estimates for each experimental protein interaction in terms of probability. For example, a yeast-two-hybrid assay indicated that both CG2275 and CG11405 were positive [4], but had a probability of interaction of just 0.2. The conflicting data indicate questionable reliability and thus further proof of interaction is required.

We also compared our predictions with other Y2H data as well as small-scaled protein-protein interaction data presented in literatures (collected by BIND) using different lab technologies, including immunoprecipitation, protein 3D structure and affinity-chromatography. The number of Y2H interactions collected in BIND is 23,088. There are only 591 interactions not included in our original dataset. As expectancy, flyDPI hits more interactions than others when we compared the overlaps amid our predictions and other experimental methods with those interactions in Y2H from BIND. Meanwhile, the overlaps between specific experimental method and flyDPI are more than those ones between specific experiment method and Y2H from BIND (Supplement S3 and S4 in additional files 3 and 4).

The Fly-DPI assembles available information on protein functions, metabolic pathways, and gene expressions from Gene Ontology (GO), GenBank, InterPro, FlyBase, KEGG, and Unigene. This biological information is useful for assigning possible functions to an unknown protein. For example, if a protein is identified as interacting with proteins involved in a particular pathway, this strongly indicates that it plays a role in that particular pathway, or may even perform the same biological functions with its partners. The EST expression data from Unigene provides insights regarding the location and the time span expressed by a gene, which can then be used to identify the protein it encodes. Proteins that are not expressed simultaneously have little chance of interacting with each other. Consequently, information on gene expression itself serves as a strong biological filter for the reliability of protein-protein interaction. The space-temporal or stage-specific expression patterns of genes are also important keys for identifying proteins involved in particular situations. The use of adequate filtering strategies can help researchers to obtain a bird's eye view of the network of interest.

The identification of potential pathways is an important objective in systems biology. The innovative strategy of ping-pong search is a naïve path map retrieval tool for fly interactome based on the shortest paths between any two proteins collected in Fly-DPI. This method localizes putative pathways including both proteins and provides further insight regarding their interaction with their partners. For instance, if two membrane receptors are chosen as the query seeds, the common signaling effector of the two signal pathways may be identified as one node in the searching result. The smart interactive interface can enable users to identify the paths between any two target proteins with options to refine the paths between them. This study also analyzes the network properties of fly interactome. This investigation found that the average shortest path lengths between any two proteins are 3.78 (all interactions) and 4.06 (interactions with high confidence), respectively (Supplemental data S2 in additional file 2). These short path lengths suggest that a biological system prefers a rapid response mechanism with low energy costs.

## Conclusion

Proteins control and mediate many biological activities of cells via interactions with other protein partners. To understand how a cell behaves and the consequent phenotype it exerts, protein networking information derived from protein interactions can serve as a starting point for exploring the cell machinery. The Fly-DPI provides an integrated proteomics tool with comprehensive and useful protein annotation information from major databases and a system for predicting protein-protein interactions. From a systems biology perspective such an integrated database should be able to reduce survey times (e.g., identify an appropriate protein target) and reduce wastage of laboratory resources (including time, labor, and expenses).

First, to deal with the false-positives resulting from high-throughput Y2H experiments, the Fly-DPI offers two referencing systems, statistical and biological, to assess data reliability. The hybrid model provides statistical estimation of the probability of putative protein interaction relationships from the domain-domain interactions decomposed from the experimental data. The biological annotation helps researchers assign functions to unknown proteins based on their interacting partners. Furthermore, the ping-pong search tool provides a naïve path map between two chosen proteins via pre-computed shortest paths from the Fly-DPI. The ping-pong search behaves more like an intuitive graphic tool to help discover potential paths within a pathway or in the convergence of different pathways. Fly-DPI is maintained as an updated interactome database by routinely renewing the annotation databases and incorporating newly published interactions into the system. Other sophisticated and advanced statistical models, such a weighting system, are evaluated to improve prediction accuracy and provide a rewired database integrated with experimental and predicted interactome within a systems biology perspective.

## Implementation and data sources

### Data sources and annotation

Protein-protein interaction data of *D. melanogaster *proteomics used in this study were obtained from three recently published high-throughput yeast two-hybrid experiments [3,36,37] and the collection of some other experiments in FlyBase Gene Annotation reports [38]. The total dataset sets (23,802 non-redundant interactions derived from 15,444 proteins) and the high-confidence dataset (2776 non-redundant interactions derived from 1850 proteins interactions) were used as our starting datasets.

Domains are recognized as functional blocks of compact protein structures, which are frequently lineated in a cassette-like fashion. They are usually evolutionarily conserved and contribute to versatile functions of a protein [39]. Therefore, we built a statistic model to predict protein interaction networks based on insights into domain interactions. We first enumerated the co-occurrence of domains in the 23,802 interactive events. Accordingly, the protein interactive data were converted into a probability score of domain interactions. These scores were then employed to infer putative interacting partners among all of the annotated open reading frames (ORFs) of *D. melanogaster *as described below.

Annotations for each protein and domain in the Fly-DPI were obtained from GO (version 1.303, 12/19/2005), FlyBase (release 4.2), Unigene (build 38), UniPort Knowledgebase (Release 6.4, 11/8/2005) [40], Integr8 Release 21 (7/11/2005) [41], and KEGG (7/10/2005).

### Statistical model: hybrid model of the association and Maximum Likelihood Estimation (MLE) methods

The statistical model of the protein interaction network is built by extracting the chance of the co-current of two domains in an interaction from the protein interaction dataset. Two proteins (*Pi, Pj*) interact (*Pij = 1*) means at least one pair of their domains interacts to each other. The probability of each pair of domains having an interaction, Pr*ob*(*D*_*mn *_= 1), is then estimated, where *D*_*mn *_= 1 if domain m (*D*_*m*_) interacts with domain n (*D*_*n*_) while *D*_*mn *_= 0 if these two domains does not interacts with each other.

Our Hybrid Model assumes that (1) two proteins (*Pi, Pj*) have interaction (*Pij = 1*) if at least one pair of their domains interacts; (2) any pair of domains having interaction is presumed to be an independent event from others. The first assumption is obvious under current knowledge to protein-protein interactions. The second assumption may be obscure if several domains are found to have similar occurrence patterns from the same protein-protein interaction data. However, it is biologically reasonable to assume the independence of domain interaction.

Therefore, the probability that a specific pair of proteins having interaction is



where *D*_*mn *_is a set of two domains {*D*_*m*_,*D*_n_} from {*P*_*i*_,*P*_j_} and *D*_*mn *_= 1 indicate the interaction between *D*_*m *_and *D*_*n*_.

Regarding to the chance of domain pairs co-currence in the entire protein-protein interaction network, the interactome, we use the likelihood function *L *to calculate all potential protein-protein interactions:



where *O*_*ij *_is a binary element indicating the status of the observed interaction. The probability of observing a pair of protein-protein interaction is constructed with pre-estimated false positive rate *fp *and false negative rate *fn *by:

Pr(*o*_*ij *_= 1) = Pr(*P*_*ij *_= 1)(1 - *fn*) + (1 - Pr(*P*_*ij *_= 1))*fp*

Based on the previous study, f_n _is near 0.80 and f_p _is estimated to be <3.6 × 10–4 [42]. A general approach, maximum likelihood estimation (MLE) method with EM algorithm [11] is utilized to estimate the probabilities Pr(*P*_*ij *_= 1) in the likelihood function. The maximum likelihood estimates are obtained to maximize the likelihood function *L*. However, the high dimensionality of the parameters introduces the computational difficulty. In addition, a calculation starting from different initial values of *D*_*mn *_may encounter the local maximal peak value which could be far from a good estimate. It is crucial to choose adequate initial values to ensure our result is biological relevance. Therefore, we applied **association measure **[12] as the initial values in EM iterations.

The association measure is the ratio between the frequency of a domain pair found in the observed protein-protein interactions and that of a whole network. A high association measure indicates the domain pair occurs in many interacting protein pairs. The explicit form of the association measure is given as



*I*_*mn*_: the number of interacting protein pairs containing the domain pair (*D*_*m*_, *D*_*n*_).

*N*_*mn*_: the total number of protein pairs containing (*D*_*m*_, *D*_*n*_)

Hybrid model provides an estimated probability of each domain pair to interact. By selecting an appropriate threshold, all estimated probabilities are dichotomized as predictors of protein interactions. A higher threshold will restrict the prediction with higher confidence of the predicted protein interactions.

The accuracy of the prediction is estimated by the ***Specificity ***and ***Sensitivity. Sensitivity ***is calculated by dividing the number of true positives (TP) through the number of all positives, which equals the sum of the true positives and the false negatives (FN); ***specificity ***is calculated by dividing the number of true negatives (TN) through the number of all negatives, which equals the sum of the true negatives and the false positives (FP). Because of the interdependency of parameters, the complexity in searching for global optimization increases the computational difficulty. There is no complete computation reported in related publications yet, while only stepwise interactions have been approached.

## Authors' contributions

All authors have contributed together towards this goal. CL conceived of this study, integrated all the information, sketched the succinct web design and drafted the manuscript. SC participated in manuscript preparation, discussions and provided many useful suggestions for web interface and presentation. PC carried out a major part of the work including developing of statistical model and manuscript preparation. CC (Chi-Shiang Cho) devoted a lot of time in implement whole infrastructure including the design on schema of database, web GUI for dynamic network, robustness and security of Linux -Apache- Mysql -PHP platform. FL helped in revising the website, updating of database with annotations and implement of hybrid model for prediction. CL (Chieh -Hwa Lin) and CC (Chia-Ling Chen) helped in data collections from various sources, compared the predictions with experimental datasets and update of databases. CL (Chen-Zen Lo) participated in processing information from EST, KEGG and GO into structural way for web presentation and biologic filters. CAH supervised and directed the development process of the whole project and guided in statistical analysis, conforming to ethical principles, critical examination and manuscript preparation. All authors have read and approved the final manuscript.

## Supplementary Material

Additional File 1 To apply Jack-knife method in the validation, in each time test set is re-sampled and the average sensitivity and specificity are calculated. The ratio between the size of the training set and test set is set to be 9:1. Click here for file

Additional File 3 Comparison with all dataset existed in BIND with Fly-DPI (all). Click here for file

Additional File 4 Comparison with all dataset existed in BIND with Fly-DPI (high confidence). Click here for file

Additional File 2 Distribution of the shortest path between pairs of proteins in Fly-DPI. On average, any two proteins in the network are connected via 3.78 (all interactions) and 4.06 (interactions with high confidence) in our data, respectively. Click here for file
