# Supplementary material for: Fly-DPI: database of protein interactomes for D. melanogaster in the approach of systems biology
Source: BMC Bioinformatics. 2006 Dec 18;7(Suppl 5):S18. doi: 10.1186/1471-2105-7-S5-S18 (PMC1764474; doi:10.1186/1471-2105-7-S5-S18)

Supplemental data S1: To apply Jackknife method in the validation, in each time test set is re-sampled and the average sensitivity and specificity are calculated. The ratio between the size of the training set and test set is set to be 9:1.


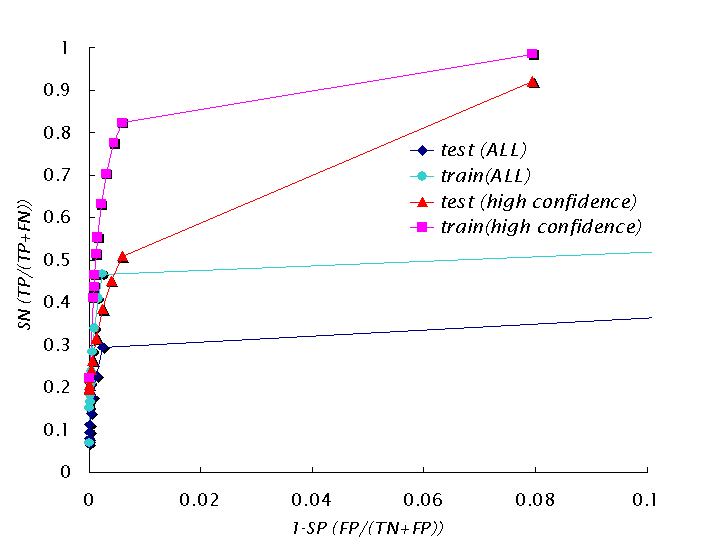

Supplement: Additional File 1 — To apply Jack-knife method in the validation, in each time test set is re-sampled and the average sensitivity and specificity are calculated. The ratio between the size of the training set and test set is set to be 9:1. [file 1471-2105-7-S5-S18-S1.doc]
