# Supplementary material for: Fly-DPI: database of protein interactomes for D. melanogaster in the approach of systems biology
Source: BMC Bioinformatics. 2006 Dec 18;7(Suppl 5):S18. doi: 10.1186/1471-2105-7-S5-S18 (PMC1764474; doi:10.1186/1471-2105-7-S5-S18)
Supplement: Additional File 3 — Comparison with all dataset existed in BIND with Fly-DPI (all). [file 1471-2105-7-S5-S18-S3.doc]

Supplemental data S3: Comparison with all dataset existed in BIND with Fly-DPI (all).

|  | Total number of protein interactions | Number of hits to Fly-DPI | Overlaps with Y2H in BIND (23088) | not R* is P^#^ | is R not P | is R and P |
| --- | --- | --- | --- | --- | --- | --- |
| affinity-chromatography | 166 | 25 | 0 | 12  (5 prob>0.6 in which 3 prob=1) | 7  (all prob<0.2) | 6  (3 prob>=0.4 in which 1 prob=1) |
| immunoprecipitation | 125 | 15 | 14 | 7  (4 prob>0.4 in which 3 prob=1) | 6  (all prob>0) | 2  (1 prob=1) |
| three-dimensional structure | 67 | 18 | 0 | 12  (5 prob=1) | 0 | 6  (all prob=1) |
| Y2H (except our datasets) | 575 | 81 | / | 31  (13 prob>=0.4 in which 10 prob=1) | 27  (all prob>0) | 23  (15 prob>=0.4 in which 14 prob=1) |

*R: This interaction is listed in specific experimental method.

^#^P: This interaction is predicted by our model.
