# Supplementary material for: Fly-DPI: database of protein interactomes for D. melanogaster in the approach of systems biology
Source: BMC Bioinformatics. 2006 Dec 18;7(Suppl 5):S18. doi: 10.1186/1471-2105-7-S5-S18 (PMC1764474; doi:10.1186/1471-2105-7-S5-S18)
Supplement: Additional File 4 — Comparison with all dataset existed in BIND with Fly-DPI (high confidence). [file 1471-2105-7-S5-S18-S4.doc]

Supplemental data S4:. Comparison with all dataset existed in BIND with Fly-DPI (high confidence).

|  | Number of protein interactions | Number of hit to Fly-DPI |
| --- | --- | --- |
| affinity-chromatography | 166 | 21 |
| Immunoprecipitation | 125 | 11 |
| three-dimensional-structure | 67 | 16 |
| Y2H (not include 3 core datasets) | 575 | 59 |
