# Supplementary material for: Fly-DPI: database of protein interactomes for D. melanogaster in the approach of systems biology
Source: BMC Bioinformatics. 2006 Dec 18;7(Suppl 5):S18. doi: 10.1186/1471-2105-7-S5-S18 (PMC1764474; doi:10.1186/1471-2105-7-S5-S18)
Supplement: Additional File 2 — Distribution of the shortest path between pairs of proteins in Fly-DPI. On average, any two proteins in the network are connected via 3.78 (all interactions) and 4.06 (interactions with high confidence) in our data, respectively. [file 1471-2105-7-S5-S18-S2.doc]

Supplemental data S2: Distribution of the shortest path between pairs of proteins in Fly-DPI. On average, any two proteins in the network are connected via 3.78 (all interactions) and 4.06 (interactions with high confidence) in our data, respectively.
